# Glycine-Induced Hypo-Osmolar Hyponatremia and Hyperammonemia: A Case Report of Operative Hysteroscopy Intravascular Absorption (OHIA) Syndrome

**DOI:** 10.7759/cureus.70176

**Published:** 2024-09-25

**Authors:** Namrah Hameed, Sharanya Suresh Kumar

**Affiliations:** 1 Intensive Care Unit/General Practice, Thumbay University Hospital, Ajman, ARE

**Keywords:** cerebral edema, fatal, glycine, hyperammonemia, hyponatremia, leiomyoma, polyuria, seizure

## Abstract

We discuss a case of a 35-year-old female with no known medical conditions, who developed symptoms of confusion, disorientation, and seizures after an uncomplicated hysteroscopic myomectomy of her uterine fibroid using 1.5% glycine solution under general anesthesia (GA). Thanks to prompt diagnosis, management, and periodic follow-up of the patient, her symptoms resolved within 72 hours after the involvement of a multidisciplinary team. Hypo-osmolar hyponatremia with hyperammonemia is a serious and fatal metabolic disturbance seen in patients with glycine intoxication and it requires urgent treatment.

## Introduction

Hysteroscopy requires the insertion of either a flexible or a rigid hysteroscopy through the cervix into the uterus, with the aid of distending media to get a complete visualization of the endometrial cavity. This is a minimally invasive procedure that is used for treating intrauterine pathologies. Electrolyte-rich liquids as distention media are not quite popular due to their risk of conducting electricity outside the surgical field [1]. Hence, for this purpose, glycine (1.5%) is one of the most commonly used solutions, as it is nonconductive and has good optical properties. However, since this is an electrolyte-free liquid, absorption of glycine can lead to various electrolyte imbalances such as hyponatremia, hypokalemia, or even hypo-osmolality that can lead to cerebral edema or pulmonary edema [2].

A fluid deficit upper limit of 1000 ml is advised when using a hypotonic solution as the distention media, while for an isotonic solution, 2500 ml is recommended when using as a distention fluid for the procedure. However, the fluid deficit cut-off is lower for elderly patients and those with pre-existing medical conditions [1]. It has been observed that operative hysteroscopies are more prone to intravascular absorption syndrome when compared to transurethral resection of the prostate syndrome (TURP) due to differences in their irrigation pressure and fluid absorption properties [3]. We present a case of a 35-year-old female who developed acute symptomatic hyponatremia causing cerebral edema secondary to hysteroscopic removal of her uterine myoma using a 1.5% glycine solution.

## Case presentation

The patient was a 35-year-old female with no known medical conditions who presented to the Obstetrics and Gynecology ER with chief complaints of prolonged vaginal bleeding in the past. A pelvic ultrasound revealed a well-defined mixed echogenic lesion in the endometrial cavity suggestive of uterine fibroid intracavitary measuring around 4.2 x 3.6 x 3.6 cm. There were no other symptoms or pathological findings. She had no known allergies. The patient was scheduled for hysteroscopic myomectomy under general anesthesia (GA) for which she gave written consent.

Preoperative laboratory findings, which included the CBC, electrolytes, and coagulation parameters, showed a low-normal level of hemoglobin and elevated WBC counts while other labs were within normal range. Before the induction of anesthesia, the patient's vitals were stable. GA was induced with propofol and fentanyl. Cisatracurium was used to facilitate endotracheal intubation. Anesthesia was maintained with oxygen, air, and sevoflurane. She was hemodynamically stable throughout the procedure. Hysteroscopic resection of the uterine fibroid was performed successfully in two hours and the tissue was sent for histopathological examination. A total of 2 liters of glycine 1.5% was used as the distension medium. She was extubated at the end after the reversal of the neuromuscular blockade with neostigmine and glycopyrrolate and was shifted to the recovery room and then to the Obstetrics and Gynecology ward.

At three to four hours post-surgery, the patient became restless and agitated. She was found to have altered mental status with abnormal involuntary jerky movements followed by two episodes of seizure. The patient was immediately managed with antiepileptics, i.e., levetiracetam and midazolam, and was subsequently shifted to the ICU for further workup and management.

**Figure 1:**
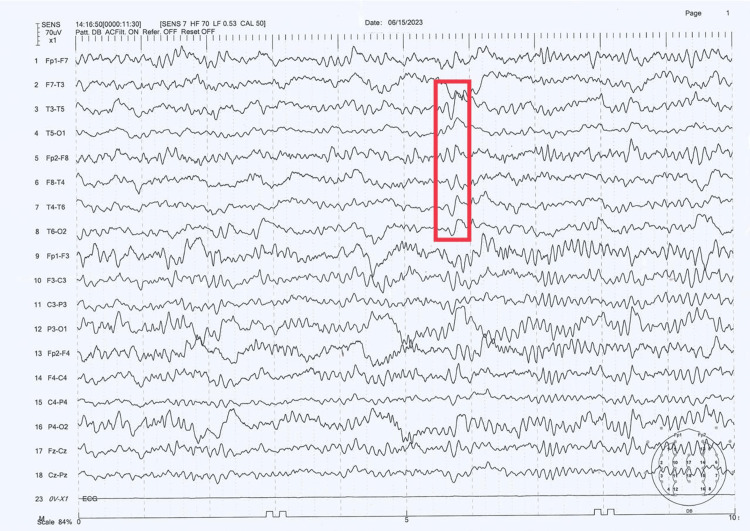
EEG performed on June 15, 2023 The results show anteroposterior phase lag with triphasic wave morphology suggestive of metabolic/toxic encephalopathy EEG: electroencephalogram

Serum electrolytes analysis revealed hyponatremia with decreased serum and urine osmolality along with hyperammonemia. Venous blood gas analysis showed features of metabolic acidosis (bicarbonate deficit of 305.2 mmol). The patient’s procalcitonin level was elevated and she was subsequently started on ceftriaxone and metronidazole, after which her procalcitonin level decreased in 24 hours. An EEG was done, which showed generalized epileptiform abnormalities admixed with occasional triphasic waves (Figure 1), suggestive of metabolic/ toxic encephalopathy. A multidisciplinary team comprising internists, a neurologist, an obstetrician-gynecologist, and a nephrologist was involved in her care. She was immediately started on 3% hypertonic saline infusion (1-2 ml/kg/hr) with the goal of increasing serum sodium level of 6-8 mEq/L in the next 24 hours (keeping in mind not to exceed 10-12 mEq/L). Lactulose was added to her regimen for the management of hyperammonemia.

The patient’s electrolytes were initially measured every eight hours. Once an improvement was noted, it was performed every 24 hours. During her stay in the ICU, the patient developed significant polyuria up to 300-400 ml/hr, which improved with the care she received in the ICU. Repeat electrolytes showed normal serum sodium levels and reduced serum ammonia levels. Table 1 presents the patient’s laboratory parameters during her stay in the ICU.

**Table 1:** Patient's laboratory parameters

Timing		Comple blood count			Electrolytes				Urine osmolality (300-900 mOsm/kg)	Serum osmolality (275-295 mOsm/kg)	Serum ammonia (11-51 umol/l)
		Hemoglobin (13-17 g/dL)	Platelet (150-410 x 10^3^/uL)	WBC (4.0-10.0 x 10^3^/uL)	Na (136-145 mmol/L)	K (3.5-5.1 mmol/L)	Cl (98-107 mmol/L)	Bicarb (21-31 mmol/L)			
												Preop		11.6	184	22.5	138	3.9	102	27	N/A	N/A	N/A
Postop	Hour 0	12.1	259	25.6	122.2	4.2	87	13.1	50.3	262.1	132
												Hour 8	N/A	N/A	N/A	137.4	3.6	107	23	286.6	N/A	N/A
	Hour 16	10.5	156	15.6	138.5	3.4	108	20.2	N/A	N/A	36											
												Hour 40	9.8	144	11.9	134.7	3.5	105	22.6	N/A	N/A	N/A

**Figure 2:**
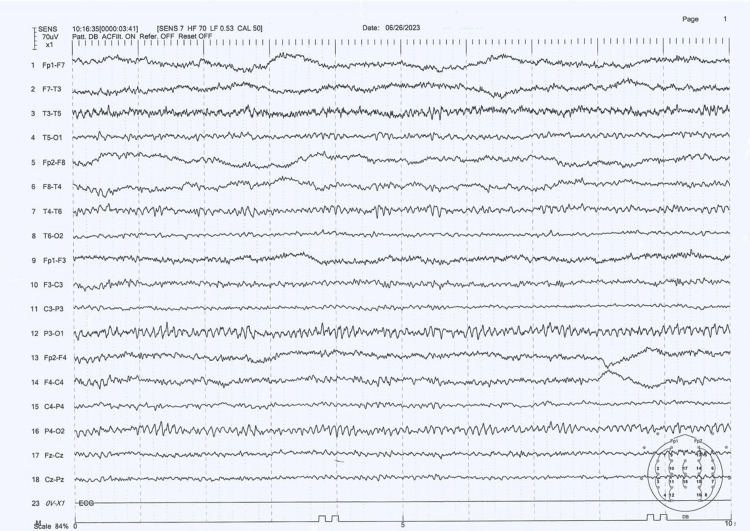
Follow-up EEG performed on June 26, 2023 The results show the resolution of triphasic waves (normal EEG) EEG: electroencephalogram

The patient was stepped down to the ward on day three and was later discharged after two days. She was stable at the time of discharge and was followed up after 10 days at the Neurology clinic and was found to be well. Figure 2 represents the patient’s EEG tracings recorded during her follow-up. Her biopsy report revealed leiomyosarcoma, for which she is currently seeking treatment in her home country.

## Discussion

Glycine is a simple amino acid that is usually mixed in water as a 1.5% solution. It is electrolyte-free and is hypo-osmolar at 200 mOsm/L. Intravascular absorption of this solution may cause water intoxication with hypervolemia and hyponatremia, which are encountered in up to 2% of TURP procedures. Normal saline is another liquid used as a distension medium, and, since it is isotonic, it is usually not associated with hypo-osmolality or electrolyte imbalances [2]. Glycine solution may have access to the vascular space when large uterine vessels are transected during surgery. Fluid under pressure in a distended uterine cavity can rapidly pass into the vasculature. Serum sodium level decreases as this fluid enters the circulation. Under normal conditions, sodium and its associated anions account for the majority of its plasma osmolarity. A rapid decrease in serum sodium usually reflects a rapid drop in serum osmolarity. However, the osmotic activity of the absorbed glycine molecules initially helps maintain serum osmolarity.

Unfortunately, glycine does not remain in the intravascular space as its half-life is only 85 minutes, after which it is absorbed intracellularly. A large intravascular bolus of this irrigation fluid, which commonly occurs during longer operations and extensive tissue resection, eventually leads to a surplus of free water. The failure to rapidly remove this free water would result in hypo-osmolar hyponatremia. There is often a delay in the diuresis of these patients who undergo surgeries due to the release of ADH hormone due to the stress of the surgery. Hormones, particularly progesterone, are shown to have an effect on the sodium-potassium adenosine triphosphatase enzyme, rendering women more prone to experience the symptoms of hyponatremia [4].

Studies have shown that the type of anesthetic agent used for hysteroscopic surgeries can have a role in operative hysteroscopy intravascular absorption (OHIA) syndrome. A randomized prospective study by Goldenberg et al. showed that epidural anesthesia showed a significantly higher rate of fluid absorption when compared to GA [5]. On the other hand, a randomized controlled trial conducted by Bergeron et al. reported that GA is associated with a higher rate of absorption of glycine and a faster rate of decrease in its serum sodium level (≥10 mEq/L), leading them the recommend the use of regional anesthesia for hysteroscopic surgeries [6]. Atieh et al. reported the case of a 43-year-old female who underwent a hysteroscopic removal of uterine fibroid with an endometrial ablation under GA; the patient developed acute symptomatic hyponatremia with respiratory distress and vision disturbance in the immediate post-surgical period after using 1.5% glycine [5]; in our patient, the symptoms developed a few hours after the surgery. 

Besides the complications caused by hyponatremia and hypo-osmolality, the metabolic by-products of glycine also have effects and complications on the body. Oxidative deamination of glycine in the liver and kidneys results in the buildup of glyoxylic acid and ammonia. Glyoxylic acid is then further processed to oxalate, which can then form crystals in the urine [4]. Dohrenwend and Shih reported the development of severely elevated ammonia levels of 1,592 µmol/L along with hyponatremia in a 76-year-old female who received 24 liters of 1.5% glycine for her transurethral resection of her bladder tumor (TURBT). During surgery, her bladder was ruptured with the extravasation of a large amount of glycine fluid inside her peritoneal cavity [7]. Furthermore, Karci and Erkin reported a case of a 27-year-old female who developed transient blindness and visual disturbance that persisted for about 20 hours after hysteroscopic myomectomy under GA. The possible toxic effects of irrigation solutions were considered as a possible cause of the adverse event [8]. Our case illustrates that even a mild increase in serum ammonia level may cause neurological derangement, a point that makes our case different from other published reports.

Hypertonic saline is the most commonly indicated therapy for symptomatic hyponatremia with reduced serum osmolality or cerebral edema. Initially, a 100 ml bolus of 3% hypertonic saline is given, which should provide 51 mEq of sodium and raise the serum sodium concentration by 2-3 mEq/L [9]. Repeated boluses of 100-150 ml 3% hypertonic saline can be given at 10-minute intervals for the treatment of hyponatremic encephalopathy [10]. Studies in the literature advocate against attempting a rapid increase in serum sodium levels. Initially, the serum sodium can be increased by 1-2 mEq/L per hour in the first one to two hours. An increase in serum sodium level by 5-10% has a large impact on cerebral edema [4]. European and American Colleges of Obstetricians and Gynecologists guidelines recommend against serum sodium levels exceeding by 10 mEq/L and 12 mEq/L, respectively in the first 24 hours of correction. An increase of 4-6 mEq/L in the first few hours is advised to alleviate the symptoms and cerebral herniation [11].

Furosemide 40 mg IV needs to be administered only if there are features of pulmonary edema caused by transient hypovolemia by glycine. Since loop diuretics worsen hyponatremia and hypotension, it is not recommended in hemodynamically unstable patients. Mannitol 20% is another treatment shown to be superior to loop diuretic as it is an osmotic diuretic and excretes less sodium in the urine [12]. Once an adequate intravascular volume is achieved and serum sodium levels start increasing, hypertonic saline can be replaced with normal saline infusion. The rate of infusion depends on the patient’s diuresis and sodium levels [4]. As per the US and European guidelines, short-term parenteral administration of desmopressin is done to control overcorrection and prevent water diuresis [11].

## Conclusions

Despite significant developments in the field of hysteroscopic surgery in the Gynecology and Urology departments, the appropriate choice and safe use of a uterine distension medium remain the most significant challenge. In surgical resection of large myomas or multiple tumors of the uterus, bladder, or prostate, the procedure can be done in two steps to reduce the risk of patient exposure to large amounts of distension fluid. Acute hyponatremia is a serious problem that must be recognized and treated promptly. In the case of glycine, ammonia toxicity may also complicate postoperative recovery, but rapid detection and correction with hypertonic saline along with supportive management aids in the stabilization of the patient.
